# The study of muscle remodeling in *Drosophila *metamorphosis using *in vivo *microscopy and bioimage informatics

**DOI:** 10.1186/1471-2105-13-S17-S14

**Published:** 2012-12-07

**Authors:** Rambabu Chinta, Joo Huang Tan, Martin Wasser

**Affiliations:** 1 Live-Cell Imaging and Automation of Image Analysis Group, Imaging Informatics Division, Bioinformatics Institute (BII), Agency for Science, Technology and Research (A*STAR), Singapore; 2 Department of Biological Sciences, National University of Singapore (NUS), Singapore

## Abstract

**Background:**

Metamorphosis in insects transforms the larval into an adult body plan and comprises the destruction and remodeling of larval and the generation of adult tissues. The remodeling of larval into adult muscles promises to be a genetic model for human atrophy since it is associated with dramatic alteration in cell size. Furthermore, muscle development is amenable to 3D in vivo microscopy at high cellular resolution. However, multi-dimensional image acquisition leads to sizeable amounts of data that demand novel approaches in image processing and analysis.

**Results:**

To handle, visualize and quantify time-lapse datasets recorded in multiple locations, we designed a workflow comprising three major modules. First, the previously introduced TLM-converter concatenates stacks of single time-points. The second module, TLM-2D-Explorer, creates maximum intensity projections for rapid inspection and allows the temporal alignment of multiple datasets. The transition between prepupal and pupal stage serves as reference point to compare datasets of different genotypes or treatments. We demonstrate how the temporal alignment can reveal novel insights into the *east *gene which is involved in muscle remodeling. The third module, TLM-3D-Segmenter, performs semi-automated segmentation of selected muscle fibers over multiple frames. 3D image segmentation consists of 3 stages. First, the user places a seed into a muscle of a key frame and performs surface detection based on level-set evolution. Second, the surface is propagated to subsequent frames. Third, automated segmentation detects nuclei inside the muscle fiber. The detected surfaces can be used to visualize and quantify the dynamics of cellular remodeling. To estimate the accuracy of our segmentation method, we performed a comparison with a manually created ground truth. Key and predicted frames achieved a performance of 84% and 80%, respectively.

**Conclusions:**

We describe an analysis pipeline for the efficient handling and analysis of time-series microscopy data that enhances productivity and facilitates the phenotypic characterization of genetic perturbations. Our methodology can easily be scaled up for genome-wide genetic screens using readily available resources for RNAi based gene silencing in *Drosophila *and other animal models.

## Background

The fruit fly *Drosophila melanogaster *is a well-established model to investigate the function of genes involved in human diseases, including cancer, developmental disorders and neuromuscular diseases [1,2]. Metamorphosis describes the phase in the development that lasts 5-6 days and transforms the larva into an adult fly [3]. Conversion of the body plan involves elimination of obsolete larval and the formation of adult tissues that either originate from the proliferation and differentiation of stem cells or the remodeling of larval cells. Larval muscles undergo two fates during metamorphosis. While most are destroyed by autophagic cell death [4], such as the dorsal external oblique muscles (DEOMs), a few change morphology and acquire a new function. For example, the dorsal internal oblique muscles (DIOMs) of the abdomen become temporary adult muscles [5]. The remodeling of the DIOMs involves a 4-5 fold reduction in diameter in first two days of the pupal stage and 2-3 fold increase in the last day of metamorphosis [6]. Thanks to a transparent cuticle and an increasing availability of fluorescent reporter proteins [7], the dynamics of muscle remodeling can be studied by time-lapse microscopy at high cellular resolution. A better understanding of the control of muscle size is important for human health. While disuse, starvation, ageing and disease can lead to atrophy, hypertrophy, or the increase in muscle size and strength, can result from physical exercise [8,9]. Therefore, *in vivo *imaging of muscles in Drosophila metamorphosis in combination with genetics promises to be a model for studies of human genes that regulate skeletal muscle mass. In *Drosophila*, targeted gene perturbations can be induced by the UAS-Gal4 overexpression system [10] which facilitates overexpression of proteins as well as gene silencing by RNA interference [11]. Combining genetic screens with time-lapse *in vivo *imaging may lead to the detection of transient phenotypes that can be easily missed by traditional endpoint assays [12]. Moreover, observed phenotypes in endpoint assays may not be the primary effect of gene perturbation and thus lead to inaccurate interpretations.

The quantification and 3D visualization of multi-dimensional (3D+time) microscopic image data, like those of Drosophila muscles [13], require fast and accurate image segmentation methods. Over the last decade, a number of segmentation algorithms have been developed for the analysis of the time-lapse fluorescence microscopy images. These algorithms can be classified into two classes with respect to the detection model being used. The first class of algorithms [14-18] performs frame-by-frame object detection, which do not use the information about the previous or next frames. The second class of algorithms [19-22] uses level set-based approaches for object detection in time-series images. In this category, models are first fitted to image data in a given frame and then evolve by using the final results from one frame as the starting point for segmentation in the next frame. The main advantage of the model evolution approach is that all the deformed contours or surfaces in the previous frame can be directly incorporated into the segmentation of current frame. However, level set-based model evolution approaches are impractical for large-scale image segmentations as they are considered computationally expensive.

In this study, we designed a workflow for the analysis of time-lapse microscopy data in the context of muscle apoptosis and remodeling during *Drosophila *metamorphosis. We demonstrate how the module for time-series analysis can be used to characterize phenotypic changes resulting from genetic perturbations. We show that a truncated GFP tagged version of nuclear EAST protein is able to inhibit histolysis of muscle fibers. Furthermore, we developed a semi-automated 3D segmentation method that can be used to visualize and quantify the morphological changes of muscle fibers. The method incorporates an Bayesian level set-based surface evolution [23] and cell nuclei detection based on multiple level-set [24]. Most of the published studies on 3D segmentation of microscopic images have dealt with the detection of discrete objects such as cell nuclei [14,15]. Few studies have addressed the problem of segmenting multi-nucleated muscle fibers. One previous report described the segmentation algorithm for human muscles for the purpose of measuring the cross sectional area. In contrast to our work, this method was designed for cryostat sections [25]. To our knowledge, our study is the first attempt to analyze the developmental dynamics of live multi-nucleated muscles in 3D using bioimage informatics. In the future, the methodology can be applied to the phenotypic characterization of targeted gene silencing by RNA interference.

## Methods

### Microscopy

*Drosophila* muscle cells were visualized using the fluorescent fusion proteins Grasp65-GFP [26] to label the cytoplasm and Histone-2av-mKO (histone-2av C-terminally tagged with monomeric Kusabira Orange [27]) to label cell nuclei. The reporter construct was generated by cloning Histone-2av-mKO cDNA into the pUAST vector. This reporter protein will be referred to as histone-mKO. Expression of the reporter genes were activated with the help of the UAS-GAL4 system using the muscle specific driver Mef2-GAL4 [11]. UAS-Grasp65-GFP and Mef2-Gal4 were obtained from the Bloomington Drosophila Stock Center, Indiana University. As an alternative to UAS-Grasp65-GFP, we also used the reporter gene MHC-tauGFP [28]. To produce a genetic perturbation that affects muscle remodeling in metamorphosis we used a UAS -east (1-1902)-GFP transgene which expresses the first 1902 (out of 2301) amino acids of the nuclear EAST protein [29] tagged by a C-terminal GFP. Prepupae were collected with a soft brush and examined under a fluorescent stereomicroscope (Olympus MVX10, Olympus, Tokyo, Japan) to confirm stage and reporter gene expression. Specimen (up to 30 per glass-bottom dish) were transferred to a 1.5 mm glass bottom dish (MatTek, Ashland, Massachusetts), oriented with the dorsal side facing down and mounted in either 0.5% low melting (LMP) agarose or CyGel (Biostatus Ltd, Leicester, UK).

*In vivo *imaging of muscles in metamorphosis was performed on a high-speed, line scanning Zeiss 5 Live (Car Zeiss, Jena, Germany) confocal laser scanning inverted microscope equipped with a XY scanning stage. Time-lapse acquisition was carried out in multiple locations with the help of the multi-time series (MTS) macro, which saves one LSM image file per time point and location. Datasets were recorded in two different resolutions at a frame size of 1024 × 1024 pixels. Low resolution time-lapse data with stack sizes of 20-30 z-slices were recorded at 30 minute intervals for over 4 days (199 frames) using a Zeiss EC Plan-Neofluar 10 ×/0.27 objective at voxel sizes of x/y = 1.25 μm and z = 13.20 μm. High resolution time-lapse with stack sizes of 97 z-slices were acquired at 10 minute intervals for over 2 days (360 frames) using a Zeiss Plan-Apochromat 20 ×/0.8 objective at voxel sizes of x/y = 0.62 μm and z = 1.48 μm. Due to the refractive index mismatch between lens medium air (N = 1.0) and the embedding media the refractive index mismatch correction was set to 1.33 and 1.37 for 0.5% LMP agarose and CyGel, respectively.

### Image analysis workflow and 2D time-lapse visualization

**Figure 1:**
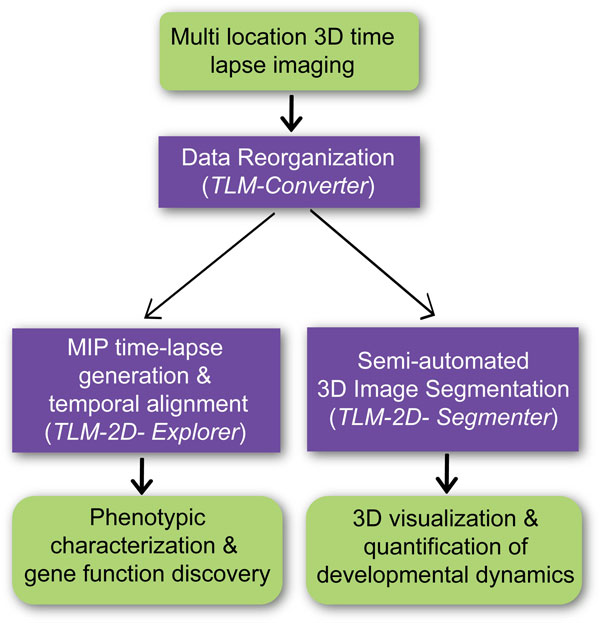
**Time-series image analysis workflow for the study of muscle remodeling in *Drosophila *metamorphosis**. (a) Upon multi-location 3D time-lapse microscopy, image data belonging to the same location are concatenated using the TLM-Converter and converted into open source ICS files. (b) The TLM-2D-Explorer produces maximum intensity projections (MIP) of 3D stacks for the rapid visual inspection of time-lapse images. Multiple MIP time-lapse datasets can be displayed side-by-side to assist in the identification of phenotypic abnormalities. Developmental reference points, such as PPT, can be used for the temporal alignment of multiple datasets. (c) To examine the morphological dynamics of individual muscle fibers in 3D, the TLM-3D-Segmenter performs semi-automated segmentation based on level sets and Bayesian surface evolution.

We designed an image analysis workflow for the analysis of multi-location 3D time-lapse datasets that consists of three components (Figure 1). During image acquisition, the Zeiss MTS macro creates one folder per location where it saves all corresponding files (1 LSM file per time point). The TLM (Time Lapse Microscopy) Converter software [30] concatenates multiple LSM files of each location into a single ICS file (The software can be downloaded from the following website: http://web.bii.a-star.edu.sg/archive/TLM-Converter). Subsequently, the 3D time-series images were transformed into maximum intensity projections (MIP) for rapid inspections using the TLM-2D-Explorer. Multiple MIP time-lapse datasets can be displayed side-by-side for phenotypic analysis. The onset of the prepupal to pupal transition (PPT), which occurs around 12 hours after pupariation, served as a reference for temporal alignment. Rotating time-series images and displaying time stamps helped to recognize phenotypic abnormalities caused by genetic perturbations and place them in the developmental context. The TLM-2D-Explorer was implemented in C++.NET with the help of the OpenCV [31] and libics [32] libraries. A third module, TLM-3D-Segmenter, performs 3D segmentation of muscle fibers (see below).

### Semi-automated segmentation of muscle fibers using TLM-3D-segmenter

**Figure 2:**
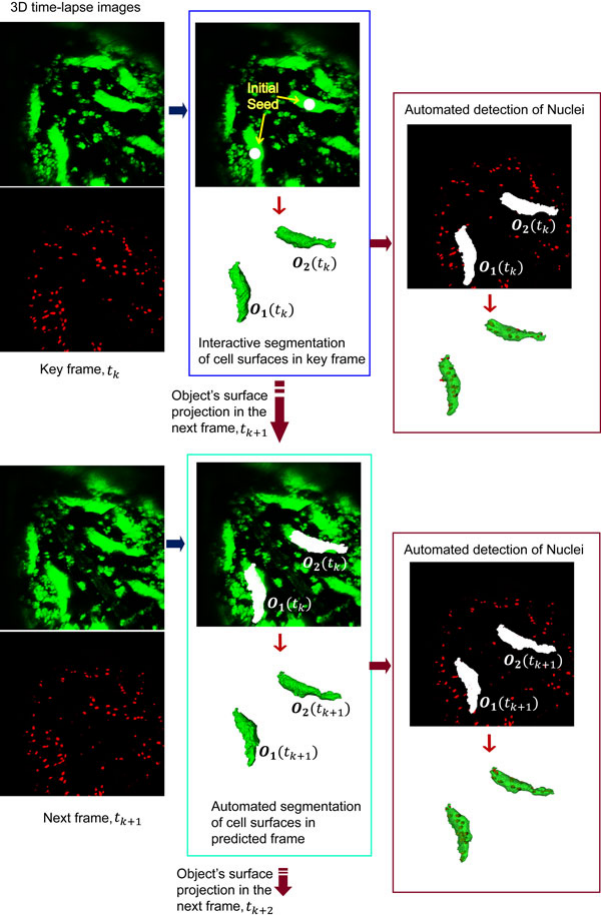
**Segmentation of muscles in 3D time-lapse Images**. Muscle cells are visualized *in vivo* using the UAS-Gal4 system with the Mef2-Gal4 driver and the fluorescent reporter proteins Grasp65-GFP (green) and Histone-mKO (red). The segmentation of 3D time-lapse image data consists of three major steps. *First*, after the user places spherical seeds in selected muscles in frame *k*, Bayesian level-set based active surface evolution detects the cytoplasmic regions (green). *Second*, the surfaces of detected objects are projected into the next frame *k+1 *to initialize and perform surface evolution of the same muscles in the next time point. *Third*, an automated 3D multi-level-set based nuclear segmentation method is applied to detect syncytial nuclei (red) within the confines of muscles cells.

We developed a semi-automatic 3D time-lapse image segmentation method for the detection of selected muscle fibers in time series image stacks. Figure 2 illustrates the three major steps of the 3D time-lapse image segmentation method. The first step consists of 3D object detection in a key frame *k*, where the user places a seed into a muscle fiber. Surface detection is performed based on level-set based surface evolution. In the second step, the converged surface is projected into the next (predicted) frame *p *where it initializes a new level set function that evolves into a new predicted surface. Propagation of the surface is repeated for a user-defined maximum number of subsequent time points. The third step detects nuclei inside the segmented muscle fibers in an automated fashion as previously described [24]. A detailed description of the 3D object detection is provided below.

### 3D object detection

In 3D object detection, we consider the case of binary partition, where a single object of interest has to be segmented from the rest of the image stack *I *= *D_I _*∈ ℝ, *D_I _*⊂ ℤ^3^. We used the concept of Bayesian region-based front evolution [33] to separate the selected object *O_i _*from background by maximizing the *a posteriori *probability (MAP) that is equivalent to minimizing the energy function. Let Φ*_i _*be the signed distance function that borders between the object *O_i _*and the background *B*. We used an implicit representation of the surface *S*(*x, y, z*) as the zero level set of a hyper-surface Φ*_i _*(*S*(*x, y, z*), *t*) [34,35]. The function Φ*_i _*defined by Φ*_i _*(X, *t*) = ± *d*, where X ∈ℝ^3 ^and *d *was the unit distance from X to the surface front *S*. The Bayesian region-based surface evolution is represented by the following equation,

(1) $$\begin{array}{c} \frac{\partial \Phi_{i}}{\partial t}\left(X\right)=-\left(\underset{\text{Dependant force, }F_{D}\left(X\right)\;}{\underset{︸}{\frac{1}{2}\left(\frac{{\left(I\left(X\right)-\mu_{B}^{}\right)}^{2}}{\sigma_{B}^{2}}-\frac{{\left(I\left(X\right)-\mu_{i}^{}\right)}^{2}}{\sigma_{i}^{2}}+4*\text{log}\left(\frac{\sigma_{B}}{\sigma_{i}}\right)\right)}}-\upsilon \underset{\text{Mean curvature, }K}{\underset{︸}{div\left(\frac{\nabla \Phi_{i}}{\left|\nabla \Phi_{i}\right|}\right)}}\right)\;\left|\nabla \Phi_{i}\right|\;\;\;\\ \;\;\;\;\;\;\;\;\;\;\;\;\;\;\;\;\;\;\;\;\;\;\;\left|\nabla \Phi_{i}\right|\to 1\;\text{for}\;\text{narrowband}\text{.}\\ ∴\;\text{The speed force, }F\left(X\right)=F_{D}\left(X\right)\;-\upsilon \;div\left(\frac{\nabla \Phi_{i}}{\left|\nabla \Phi_{i}\right|}\right) \end{array}$$

Where, $\mu_{i}=\frac{1}{\left|O_{i}\right|}\underset{\text{X}\in O_{i}}{\int }I\left(X\right)dX$ and $\sigma_{i}^{2}=\frac{1}{\left|O_{i}\right|}\underset{X\in O_{i}}{\int }I{\left(X\right)}^{2}dX-\mu_{i}^{2}$. Corresponding statistical parameters $\theta_{B}=\left\langle \mu_{B},\sigma_{B}^{2}\right\rangle$ were used for the background. *I*(*X*) denotes the intensity value of voxel *p*. The viscosity term -*υ*K helps to smooth the surface, where K is the mean curvature of surface and *υ *is the entropy condition expressing the importance of regularization.

The surface evolution algorithm is composed of two basic steps: the **initialization **and the **active surface evolution**. Initially, background and foreground regions (labeled as 0 and 1) in the entire image stack are detected as previously described [24]. Then, the level set function of a selected object is either initialized by user interaction, e.g. placing a spherical seed, or automatically with the surface of an adjacent frame. Starting from the initial surface points, recursive surface evolution is performed using queue-based data structure in breadth-first order.

#### A brief description of the algorithm is given as follows

*Initialization: *Initialize the 3D level set function Φ*_i _*= {-1, 0, 1}, -1 for the voxels of a selected seed, 0 for surface front and +1 for background. Compute the statistical parameters of background $\theta_{B}=\left\langle \mu_{B},\sigma_{B}^{2}\right\rangle$ and object regions $\theta_{i}=\left\langle \mu_{i},\sigma_{i}^{2}\right\rangle$ and initialize the queue Q by adding all the surface points of the object seed. Furthermore, *p *represents any point of the queue Q and $q\in N_{G}^{26}\left(p\right)$ any of the 26 neighborhood points of *p*.

Active Surface Evolution:

*Shrinking: *Visit all surface points in the queue Q and compute the speed *F' *of every point *p *using the following equation:

$\begin{array}{c} F^{\prime }\left(p\right)=\frac{F_{D}^{\prime }\left(p\right)}{\left|F_{D}^{\prime }\left(p\right)\right|}-\upsilon div\left(\frac{\nabla \Phi_{i}^{\prime }\left(p\right)}{\left|\nabla \Phi_{i}^{\prime }\left(p\right)\right|}\right)\\ F_{D}^{\prime }\left(p\right)=F_{D}\left(p\right)-\Delta k\frac{{\left(I\left(p\right)-\mu_{i}^{}\right)}^{2}}{\sigma_{i}^{2}},\;\Phi_{i}^{\prime }\left(p\right)=\frac{2}{\pi }{\text{tan}}^{-1}\left(\frac{\Phi_{i}\left(p\right)}{\varepsilon }\right) \end{array}$ (2)

is the regulation function and Δ*k *denotes a user-defined fine-tuning parameter for the surface evolution process that is selected based on segmentation performance compared to a manually specified ground truth as previously described [24].

If $F_{D}^{\prime }\left(p\right)$ is negative, the candidate point *p *becomes background (Φ(*p*)← 1) and all its neighboring points $\left(\Phi \left(q\right)\leftarrow 0,\;\forall q\in N_{G}^{26}\left(p\right)\;\text{and }\Phi \left(q\right)<0\right)$ are inserted into the queue Q.

*Inflation: *Visit all current surface points in the queue Q and compute the speed *F*' of every point *p *as given in Eq. (2).If $F_{D}^{\prime }\left(p\right)$ is positive, the candidate point becomes an object point (Φ(*p*) ← -1) and all its neighboring points $\left(\Phi \left(q\right)\leftarrow 0,\;\forall q\in N_{G}^{26}\left(p\right)\;\text{and }\Phi \left(q\right)>0\right)$ are inserted into the queue Q.

*Update: *Update the statistical parameters of background $\theta_{B}=\left\langle \mu_{B},\sigma_{B}^{2}\right\rangle$ and candidate object $\theta_{i}=\left\langle \mu_{i},\sigma_{i}^{2}\right\rangle$

Stopping *Criteria: *If the speed function of none of the surface front point is negative or the number of iterations reaches to pre-defined maximum, the surface evolution process is terminated. Otherwise, active surface evolution (step 2) will be continued until convergence.

### Constrained muscle detection

The inflation of active surfaces may result in segmentation errors if the image stack was recorded at a spatial resolution (e.g. using a low NA objective) that is insufficient to resolve objects in close proximity. To prevent the merging of adjacent objects that are not separated by an intensity gradient we implemented a variant of the above algorithm that differs in two aspects. First, instead of a seed, the level set function is initialized by a polygon that is manually drawn around the ROI in a projection of the image stack. As such, the polygon demarcates the maximum ROI boundary. Second, the object surface can only evolve by shrinking (the inflation operation is disabled) to constrain the surface.

### Feature extraction

Shape features were extracted to characterize the remodeling of abdominal oblique muscles during metamorphosis. Upon segmentation, 3D surfaces were projected into the xy plane to produce 2D silhouette ROIs. The tensor (2^nd ^order) moments [36,37] of the silhouette contours were calculated to derive the axis of minimum inertia, which was used to determine the mean and standard deviation of cell diameter. The extent was calculated as the ratio between the area of the ROI and the area of its minimum bounding rectangle.

### Implementation

We implemented the 3D segmentation algorithm in C++ using the OpenCV computer vision library [31], the libics v1.5.2 [38] library for Image Cytometry Standard (ICS) and the visualization tool kit (VTK) [39].The standalone application "TLM-3D-Segmenter" was developed for the Windows platform using the Qt SDK. The software is freely available for academic research upon request.

## Results

**Figure 3:**
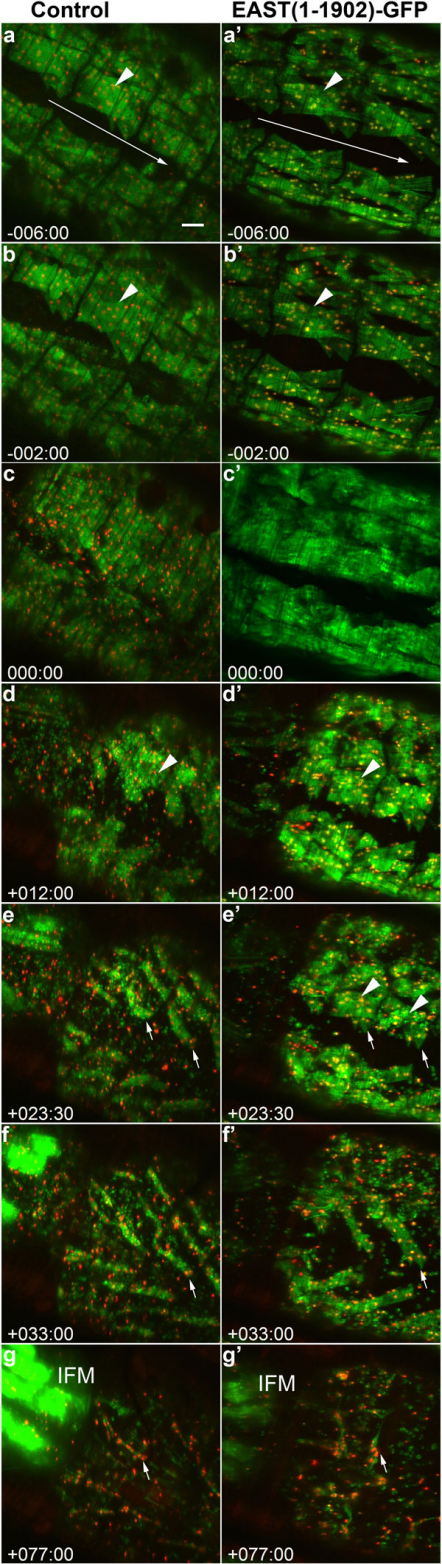
**Temporal alignments of time-series data help to uncover phenotypic abnormalities caused by genetic perturbations**. This example compares a control genotype (left panels) and a genetic perturbation caused by the overexpression of EAST(1-1902)-GFP (right panels) that interferes with muscle remodeling. The panels show maximum intensity projections (MIPs) of 3D stacks of dorsal abdominal regions. The control pupa expresses UAS-Grasp65-GFP (green) and UAS-Histone-mKO (red) driven by Mef2-Gal4 to label cytoplasm and nuclei, respectively. The *UAS-east*(1-1902)-GFP mutant pupa (green nuclei) expresses MHC-tau-GFP (green) to label cytoplasm and UAS-histone-mKO. Time stamp 0:00 (hours:minutes) refers to the onset of PPT, negative time stamps indicate prepupal and positive ones pupal stages. (a, a') At -6:00, no discernible differences are observed. The arrows point in the anterior to posterior direction along the bilateral symmetry axis. Arrow head indicate external oblique muscles that undergo apoptosis during metamorphosis. (b, b') At -2:00, deformation of the external muscle (arrow head) is more pronounced in the control than the mutant animal. (c) Muscle contractions at the onset of PPT lead to blurring of the MIPs. (d) At +12:00, disintegrating external muscles give rise to vesicles (arrow head) while the mutant specimen (d') shows intact external muscles (arrow head) and significant lower quantities of vesicles. (e) At 23:30, external muscles in the control are completely destroyed, providing a non-occluded view of the internal oblique muscles (arrows). Mutant external muscles (arrow head) show a delayed onset of muscle degeneration. Internal muscles (arrows) remain mostly occluded. (f) At 33:00, apoptosis of persistent muscles is also completed in the mutant. Note that atrophy is more advanced in the control than in the mutant (f'), resulting in a smaller diameter of the muscle fibers (arrows). (g) At 77:00, persistent control muscles (arrow) show progressive erosion and an orientation parallel to the body axis. (g') Corresponding mutant fibers (arrow) display irregular morphologies and orientations. Note that the complete time series can be viewed as a movie (Additional File 1).

To study apoptosis and remodeling of muscles in *Drosophila* metamorphosis, we designed a time-lapse image analysis system that consists of three components. The previously described TLM-Converter reorganizes multi-dimensional image data [30], the TLM-2D-Explorer is used for the exploration time-lapse data as 2D projections and the TLM-3D-Segmenter is a tool for 3D segmentation and visualization of developmental dynamics. The TLM-2D-Explorer converts 3D time-series images into maximum intensity projections that can be used for quality control of image acquisition and rapid data exploration. Multiple time-lapse datasets can be temporally aligned using developmental reference points, such as the prepupal to pupal transition (PPT), and viewed side-by-side. Browsing through equivalent developmental stages in different specimen helps to identify phenotypic abnormalities resulting from genetic perturbations. As a case study for the phenotypic characterization of a genetic perturbation, we performed an *in vivo *imaging experiment involving the overexpression of a truncated version of the nuclear EAST protein [34]. A previous study showed that the *east *(*enhanced adult sensory threshold*) gene was involved in controlling apoptosis and remodeling of various tissues in metamorphosis [21]. While overexpression of EAST-GFP was able to delay apoptosis of salivary glands, the loss of gene function resulted in premature destruction and abnormal remodeling of muscles. To evaluate our software, we induced muscle specific overexpression of a C-terminally truncated form of EAST (residues 1-1902) tagged to GFP. Full length EAST of 2301 residues could not be used as it caused developmental arrest in larvae. We performed *in vivo *imaging of the dorsal abdominal muscles in pupae over a 4-day period at 30 minute intervals. To elucidate possible phenotypic abnormalities, 192 time points starting during the prepupal stage 6 hours prior to PPT until 86 hours into the pupal stage were compared between an EAST (1-1902)-GFP overexpressing and a control sample (Figure 3). The whole time series of both samples can be viewed as QuickTime Movie (Additional File 1). During the prepupal stage (Figure 3a, a'), no discernible differences could be observed between control and genetic perturbation, indicating that the overexpression does not affect larval muscle morphology. During the early pupal stage, transgene overexpression caused a delay in the apoptosis of the DEOMs (Figure 3d-e, 3d'-e'). While the control DEOMs along the midline have completed histolysis by +12 hours (Figure 3b, arrowhead), the transgenic counterparts remain intact until 24 hours into the pupal stage. Furthermore, EAST(1-1902)-GFP overexpression was found to affect the remodeling of larval DIOMs into adult muscles. The genetic perturbation was observed to slow the reduction in the diameter of muscles fibers (Figure 3f', arrow) compared to control muscles (Figure 3f', arrow). As remodeling progressed, the truncated EAST resulted in abnormal muscle morphology followed by the collision of fibers from opposing hemi-segments (Figure 3g'). A larger C-terminal deletion EAST(1-1536)-GFP [40] did not produce any of these morphological changes (not shown), indicating that the phenotypes are specific to the EAST and not GFP portion of the transgene.

To quantify and visualize muscle remodeling in 3D, we developed a semi-automated image segmentation tool, TLM-3D-Segmenter, which is based on a previous level-set based method for the automated detection of nuclei in *Drosophila* embryos [24]. The surface of each muscle is represented by a separate level set function that evolves from a seed that the user places inside the cell body, which is visualized by the first color channel (Figure 2). Once convergence happens, the level set function of the key frame is propagated to a user-defined number of subsequent or predicted frames. Finally, nuclei are detected in the second color channel within the boundaries of the muscle surface using the above-mentioned automated nuclear segmentation algorithm. This approach was applied to monitor remodeling of a selected set of muscles in the 3D time-lapse images. We did not pursue the idea of a fully automated approach to avoid detection of incompletely shown muscles that extend out of the field of view and of GFP-labeled apoptotic cell fragments that were not the primary interest of this study.

**Figure 4:**
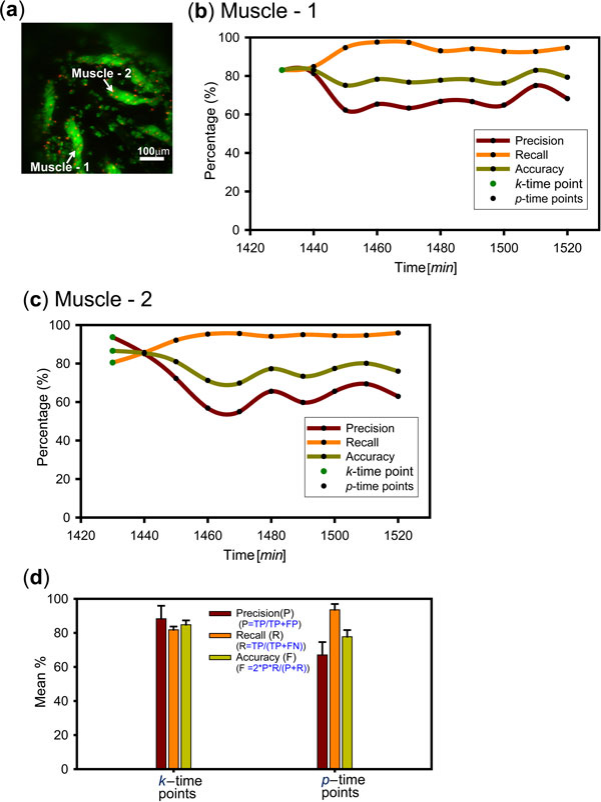
**Validation of the 3D time-lapse image segmentation method**. (a) MIP views of persistent dorsal abdominal muscles. (b) & (c) The performance of 3D image segmentation method for muscles 1 and 2 was determined for semi-automatic segmentation in frame *k *and for automated segmentation in 9 predicted frames *p*. Time-lapse data were acquired at 10 minute intervals. Time is shown relative to PPT. (d) Mean segmentation performance for the two muscles over 30 frames, including 3 *k *and 27 *p*-frames. Hence *n*_k _= 6 and *n*_p _= 54. Recall *R *= (*TP*)/(*TP-FN*), Precision *P *= (*TP*)/(*TP*-*FP*) and Accuracy F = 2*P*R/(*P+R*), with *TP *= true positives, *FN *= false negatives and *FP *= false positives.

To estimate the accuracy of image segmentation, we compared it to a manually defined ground truth for key and predicted frames. Ground truth segmentation of two muscles (Figure 4a) in 30 continuous frames was specified and verified using the ImageJ plugin "Segmentation Editor" [41]. In our tests, key frames were segmented every 10 time points and propagated forward to the next 9 frames (Figure 4b, c). Mean segmentation performance was found to be 84.0% ± 4.7% for key and 80.0% ± 6.34% for predicted frames (Figure 4d).

**Figure 5:**
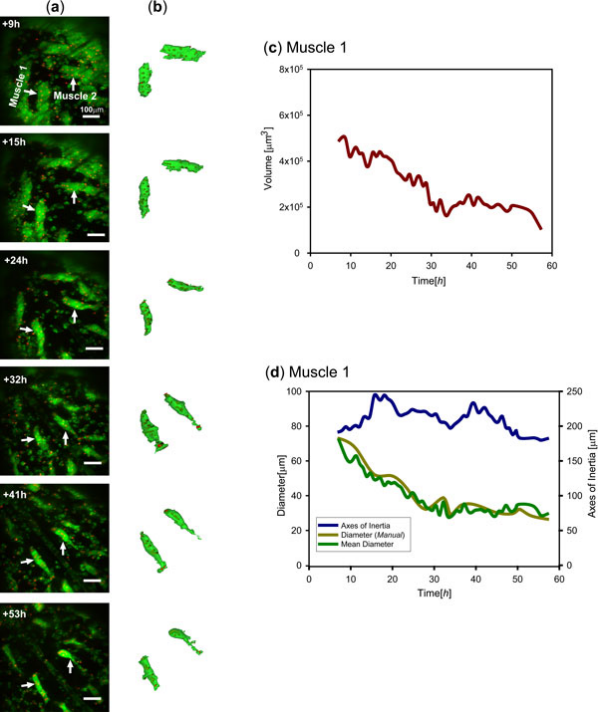
**Segmentation facilitates visualization and quantification of muscle remodeling**. (a) Selected dorsal views derived from MIPs of a pupal abdomen where muscles are labeled using Grasp65-GFP (green). Arrows indicate two segmented persistent muscles. The scale bar in the top panel represents 100 μm and applies to all other panels. (b) Iso-surface rendering of the two segmented muscles. Syncytial nuclei are rendered in red. (c, d) Segmentation permits the measurement of dynamic features, such as volume (c), diameter and the axis of inertia.

**Figure 6:**
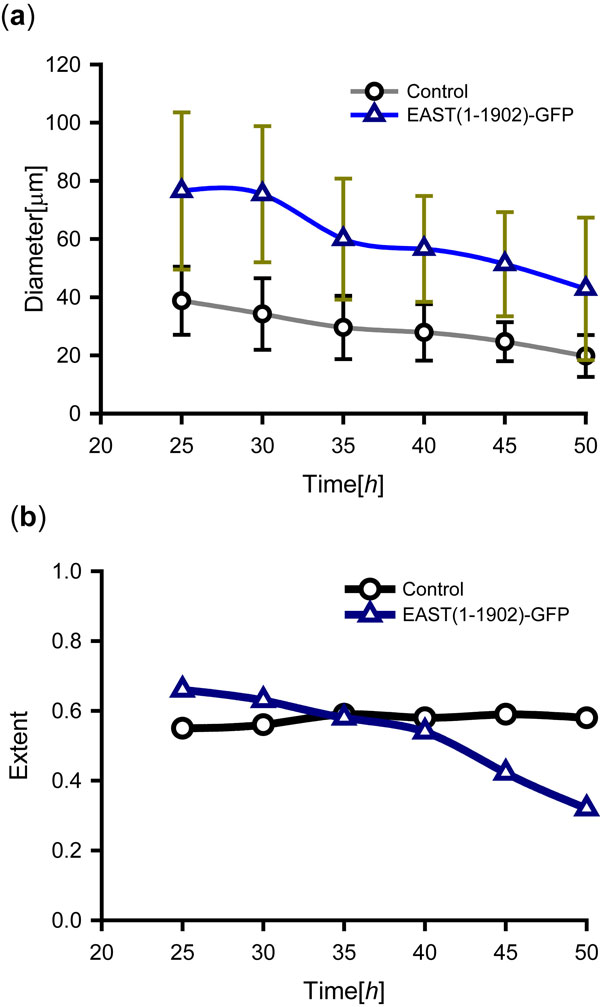
**Comparison of morphological changes during muscle remodelling between EAST(1-1902)-GFP misexpression and control genotypes**. (a) The mean diameters of silhouette ROIs of segmented muscles (Figure 3f, 3f', arrows) decrease as a result of atrophy during metamorphosis. Time is shown in hours after PPT. Consistent with a visual inspection of images (Figure 3), EAST(1-1902)-GFP misexpression leads to a delay in muscle atrophy. (b) The extent of the muscle silhouettes can be used to quantify phenotypic differences in cell shape. The progression towards irregular cell morphology is accompanied by a decrease in extent. In contrast, the corresponding values in controls remain constant while the shape of the muscle fiber maintains its straight morphology.

To examine muscle remodeling in 3D, we segmented 2 muscle fibers recorded over 50 hours at 10 minute intervals (Figure 5). Surface reconstructions of the muscle along with their syncytial nuclei provided 3D views of the cellular remodeling and the changing subcellular distributions of syncytial nuclei (Figure 5b). The segmented objects could be further used to calculate shape features such diameter and axis of inertia, to quantify the progression of muscle atrophy in metamorphosis (Figure 5c).In our experiments, we set the shift factor Δ*k *to a constant value of 0.25 based on the visual inspection of segmentation outputs. To quantify morphological differences during muscle remodeling for the mutant and control genotypes in the case study above (Figure 3), we performed 3D segmentation and extracted features from the 2D projections (Figure 6). Mean and standard deviation of the diameter along the medial axis was calculated to compare the progression of muscle atrophy (Figure 6a), while extent was determined to characterize cell shape. The time series plot confirms the observation that the overexpression of EAST (1-1902)-GFP delays the size decrease to the muscle fiber in metamorphosis. In addition, the divergence from a straight shape in the control to an irregular morphology resulting from genetic perturbation is reflected by a decreased extent (Figure 6b).

## Discussion

3D time-lapse microscopy of intact tissues is becoming an important tool to study developmental biology and the genes involved. To optimally benefit from the information hidden in large-scale digital image data, novel approaches for image processing and analysis are required. The metamorphosis of muscles and other cell types in *Drosophila *is a process that is amenable to *in vivo *imaging, cell biology and genetics. We developed a workflow that addressed a variety of challenges, including data management, intuitive phenotypic characterization and 3D visualization of cellular dynamics.

The case study illustrated the benefits of performing time-series rather than endpoint assays. Examining the terminal phenotype resulting from EAST (1-1902)-GFP overexpression at the end of metamorphosis would have uncovered an abnormal terminal phenotype of abdominal muscles. However, interpretation would be incomplete as we would have missed that the morphology was normal until the prepupal stage and that the genetic perturbation suppressed apoptosis. Metamorphosis lasts around 5 days and is dynamic. As such, quantitative and transient phenotypic effects, such as a wider diameter of muscle fibers or delayed cell histolysis are easier to detect if time-series data of control specimen of the same age are displayed in parallel. The overexpression of truncated EAST corroborates previous data of its function in inhibiting histolysis [6]. However, since endogenous *east *is expressed ubiquitously, it could be counter-argued that the muscle phenotype was a secondary effect of a different abnormality, such as loss of innervation. The targeted genetic perturbation described in this case study supports our initial conclusion. Moreover, the fact that overexpression of shorter version of GFP tagged EAST (aa 1-1536) did not affect muscle development suggests that the region spanning residues 1537-1901 may be specifically required for autophagic cell death. No defects were observed in larvae and prepupae, suggesting that EAST (1-1902)-GFP overexpression did not interfere with muscle structural maintenance and function prior to pupariation.

In the future, we plan to apply our workflow to the characterization of targeted gene silencing in muscles using RNAi. Public stock collections offer several thousand UAS-RNAi lines, e.g. the Transgenic RNAi Project (TRiP) collection [42]. Furthermore, the accuracy of the 3D segmentation requires improvements. We noticed a loss of performance of predicted frames compared to key frames segmented in an interactive fashion. The increased false positive rates could be attributed to dead muscle fragments being merged to segmented objects. Our efforts will focus on using temporal information to exclude these fragments as their mobility is higher than that of intact muscles.

## List of abbreviations used

DEOM: dorsal external oblique muscle; DIOM: dorsal internal oblique muscle; GFP: Green Fluorescent Protein; MIP: Maximum intensity projection; mKO: monomeric Kusabira Orange; PPT: prepupal to pupal transition; TLM: time-lapse microscopy.

## Competing interests

The authors declare that they have no competing interests.

## Authors' contributions

RC was involved in design and implementation of the image analysis system, performed data analysis and drafted the manuscript. JHT was involved in laboratory experiments, microscopy and image analysis. MW conceived and designed the study, was involved in design and implementation of the image analysis system, performed laboratory experiments, microscopy and data analysis, and drafted the manuscript. All authors read and approved the final manuscript.

## Supplementary Material

Additional file 1 **Temporal comparisons of time-lapse images facilitate the discovery of phenotypic abnormalities resulting from genetic perturbations**. The movie shows metamorphosis of two genotypes at 30 minute intervals, a control specimen (left panel) expressing Grasp65-GFP (green) and Histone-mKO (red) and specimen expressing EAST(1-1902)-GFP (right panel), tau-GFP (green) and Histone-mKO (red). The time-lapse starts during the prepupal stage (-6 hours) and last until late pupal stage (+88 hours). The transition from the prepupal to pupal stage begins at zero hours. Note that EAST(1-1902)-GFP overexpression lead to suppression of muscle histolysis in the first 25 hours of the pupal stage and abnormalities of muscle morphology from +38 hours onwards. See Figure 3 for more details. Click here for file
